# Synthesis of 2-(9,10-Dihydro-9,10-propanoanthracen-9-yl)-*N*-methylethanamine via a [4+2] Cycloaddition

**DOI:** 10.3390/molecules15064201

**Published:** 2010-06-09

**Authors:** Usama Karama, Adel Al-Saidey, Zeid Al-Othman, Abdel Rahman Almansour

**Affiliations:** Department of Chemistry, College of Science, King Saud University, P.O.Box 2455, Riyadh - 11451, Saudi Arabia

**Keywords:** benzoctamine, homologue, antidepressant, ring expansion, cycloaddition

## Abstract

The synthesis of the tetracyclic molecule 2-(9,10-dihydro-9,10-propano-anthracen-9-yl)-*N*-methylethanamine (**2**) as a homologue of the antidepressant 1-(9,10-dihydro-9,10-ethanoanthracen-9-yl)-*N*-methylmethaneamine (**1**) was described. The key intermediate 9-(prop-2-en-1-yl)-9,10-dihydro-9,10-propanoanthracen-12-one (**7**) was successfully synthesized via a [4+2] cycloaddition of α-bromoacrolein and 9-allyl-anthracene, followed by ring expansion and samarium diiodide deoxygenation.

## 1. Introduction

**Figure 1:**
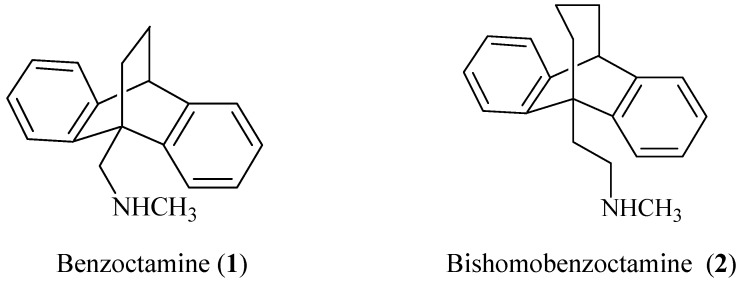
Benzoctamine and bishomobenzoctamine.

Benzoctamine [1-(9,10-dihydro-9,10-ethanoanthracen-9-yl)-N-methylmethanamine, 1, Figure 1] was synthesized and developed into a clinically useful drug for the treatment of anxiety by the Ciba-Geigy research group [1]. The key step was [4+2] cycloaddition of ethylene on 9-anthracenecarboxaldehyde.

Studies on the structure-activity relationship (SAR) of this molecules led us recently [2] to synthesize the corresponding bishomobenzoctamine, 2-(9,10-dihydro-9,10-propanoanthracen-9-yl)-*N*-methylethanamine (**2,** Figure 1)via a [3+4] cycloaddition at 15–20 ºC [3]. 

It is assumed from molecular model studies that the ring folding angle in bishomobenzoctamine **2** is different from that of benzoctamine **1**, and such a difference might be reflected in its pharmacological activities.

**Scheme 1:**
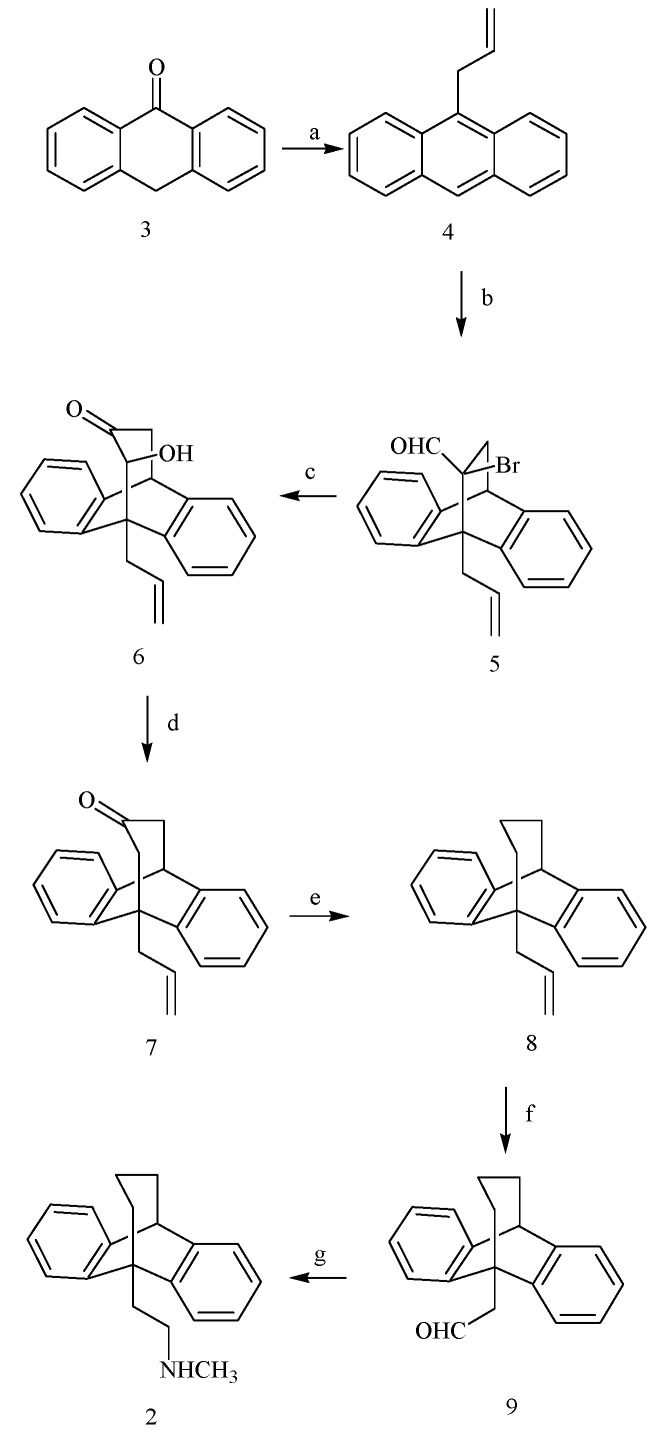
Synthesis of compound **2**. *Reagents and conditions*: a) Allylmagnesium bromide, THF, r.t., 8 h; C_6_H_6_, P_2_O_5,_ r.t., 6 h, 82%; b) α-bromoacrolein, 80 ºC, 24 h, 68.6%; c) NaOH, THF, r.t., 4 h, 60.5%; d) SmI_2_, THF, r.t., 4 h, 56%; e) 85% H_2_N-NH_2_ , KOH, triethylene glycol, 150ºC, 5h, 200–220 ºC, 5 h, 57%; f) O_3_, CH_2_Cl_2_, -78^o^C, 0.5 h; (CH_3_)_2_S, 4 h, r.t, 56%; g) CH_3_NH_2_, CH_3_OH, r.t., 4 h; NaBH_4_, r.t, 6 h, 57%.

## 2. Results and Discussion

We outline herein a simple and flexible route to the corresponding bishomobenzoctamine **2** via a [4+2] cycloaddition [4] which tolerates a variety of functional groups and is not be sensitive to high temperatures. The key intermediate 9-(prop-2-en-1-yl)-9,10-dihydro-9,10-propanoanthracen-12-one (**7**)was easily synthesized in three steps starting from 9-allylanthracene (**4**) which was obtained by the reaction of anthrone **3** with allylmagnesium bromide followed by dehydration using P_4_O_10_ (Scheme 1).

The Diels-Alder reaction between compound **4** and α-bromoacrolein afforded the cycloadduct **5**. Treatment of **5** with 1M aq NaOH resulted in transformation [5] into the ring expanded α-hydroxy ketone **6**. Deoxygenation of **6** by samarium iodide led to the desired key intermediate ketone **7**. Wolff-Kishner reduction of the ketone **7** gave the tetracyclic hydrocarbon 8, which was ozonolyzed to the crystalline aldehyde **9**. Reductive amination of the aldehyde **9** using a combination of commercially available solution of methylamine in methanol, titanium(IV) isopropoxide and sodium borohydride [6] afforded the bishomobenzoctamine **2**.

## 3. Experimental

### 3.1. General

IR spectra were recorded on a Perkin-Elmer 883 spectrophotometer and peaks are expressed as ν (cm^-1^). NMR spectra were recorded on a JEOL ECP 400 (400 MHz) instrument in CDCl_3_ and chemical shifts are expressed as δ ppm, and coupling constants (*J*) are given in Hertz. MS spectra and HRMS were performed at the Department of Organic Chemistry of the University of Hannover-Germany using EI at 70 eV. 

*9-Allylanthracene* (**4**). A solution of anthrone **3 ** (5.01 g, 25.8 mmol) in anhydrous THF (100 mL) was slowly added to allylmagnesium bromide (33 mL, 33 mol, 1 M solution, Aldrich). The mixture was stirred for 8 h at room temperature. The reaction mixture was subsequently acidified with 10% HCl, the organic layer was separated, and the aqueous layer was extracted with ether (2 × 50 mL). The combined organic layer was washed with water, dried over Na_2_SO_4_ and the solvent was evaporated. To the crude product was added 25 mL benzene, 6 g P_4_O_10_ and stirred for 6 h at room temperature. The P_4_O_10_ was filtered off and the benzene was removed under vacuum. The crude product was purified by flash column chromatography (hexane-dichloromethene 1:1) to give **4** (4.6 g, 82%) as a yellow solid, m.p. 46 ºC; IR (KBr): ν = 3047, 2945, 1620, 1444, 729 cm^-1^; ^1^H-NMR δ 4.37 (d; *J* = 5.48 Hz, 2H, H-1'), 4.97 (dd; *J* =10.24, 1.48 Hz, 1H, H-3'), 5.06 (dd; *J*= 16.84,1.48 Hz, 1H, H-3'), 6.21–6.28 (m; 1H, H-2'), 7.28–7.60 (m; 9H, aromatic -H); ^13^C-NMR δ 32.00, 116.00, 124.89, 125.36, 126.25, 128.20, 130.06, 131.56, 131.71, 134.05, 136.50. MS (EI) *m/z* (%) = 218 (100) [M^+^], 203 (54), 191 (27), 176 (5), 165 (7); HRMS (EI) Calcd. for C_17_H_14_ [M^+^] 218.1096, Found 218.1097.

*12-Bromo-9-(prop-2-en-1-yl)-9,10-dihydro-9,10-ethanoanthracen-12-carbaldehyde* (**5**). A mixture of 9-allylanthracene (**4**, 2.1 g, 9.65 mmol) and α-bromoacroline (2.61 g, 19.3 mmol) in benzene (10 mL) was heated under reflux 24 h and allowed to cool to room temperature. The reaction mixture was concentrated and the crude product was purified by flash column chromatography on silica gel (petroleum ether-ethyl acetate 30:1) to give **5** (2.33 g, 68.6%) as a white solid, m.p. 145 ºC; IR (KBr): ν = 3070, 2970, 1716, 1631, 435, 914, 748 cm^-1^; ^1^H-NMR δ 2.2 (dd; *J =* 14, 2.9 Hz, 1H, H-11), 3.01 (dd; *J =* 14, 2.9 Hz, 1H, H-11), 3.43–3.49 (m, 1H, H-1'), 3.81–3.86 (m, 1H, H-1'), 4.33 (t; *J =* 2.9 Hz, 1H, H-10), 5.27–5.31 (m, 1H, H-3'), 5.44–5.49 (m, 1H, H-3'), 5.95–6.05 (m; 1H, H-2'), 7.12–7.53 (m; 8H, aromatic-H), 9.35 (s; H-C=O); ^13^C-NMR δ 31.47, 42.13, 43.83, 52.85, 75.45, 117.89, 123.66, 125.57, 126.05, 126.95, 136.67, 139.19, 143.12, 191.04; MS (EI) *m/z* (%) = 352 (23), 354 (24) [M^+^], 274 (35), 273 (84), 272 (100), 219 (67), 218 (100), 215 (62), 204 (27), 203 (70), 202 (66), 191 (49), 178 (21), 165 (32); HRMS (EI) Calcd. for C_20_H_17_BrO, C_20_H_17_BrO [M^+^] 352.0465, Found 352.0463.

*11-Hydroxy-9-(prop-2-en-1-yl)-9,10-dihydro-9,10-propanoanthracen-12-one* (**6**). To a solution of the Diels-Alder adduct **5 **(2 g, 5.69 mmol) in THF (20 mL) was added 1M aqueous NaOH (21 mL). The mixture was stirred at room temperature for 4 h, extracted with ether twice, washed with water, dried with MgSO_4_ and concentrated under vacuum. The crude product was purified by flash column chromatography on silica gel (petroleum ether-ethyl acetate 5:1) to give **6** (1 g, 60.5%) as a white solid, m.p. 152 ºC; IR (KBr): ν = 3512, 3464, 3022, 2920, 1701, 1448, 1350, 1124, 731 cm^-1^; ^1^H-NMR δ 2.79 (dd; *J* = 15.5, 1.5 Hz, 1H, H-11), 3.15(dd; *J* = 15.5, 6.6 Hz, 1H, H-11), 3.37–3.45 (m; 1H, H-2'), 3.82–3.88 (m; 1H, H-2'), 4.03 (d; *J* = 3.5 Hz, 1H, H-O), 4.11(d; *J* = 3.5 Hz, 1H, H-10), 4.32 (dd; *J* = 6,1.5 Hz, 1H, H-9), 5.14–5.17 (m; 1H, H- H-3'), 5.35–5.39 (m; 1H, H-3'), 5.58–5.67 (m; 1H, H-2'); ^13^C-NMR δ 32.55, 43.50, 50.24, 50.32, 84.23, 119.25, 126.09, 126.49, 126.75, 127.57, 134.96, 140.25, 143.88, 208.22; MS (EI) *m/z* (%) = 290 (48) [M^+^], 218 (100), 217 (71), 215 (53), 203 (67.77), 202 (60), 191 (57), 178 (34), 152 (11); HRMS (EI) Calcd. for C_20_H_18_O_2_ [M^+^] 290.1305, Found 290.1307.

*9-(Prop-2-en-1-yl)-9,10-dihydro-9,10-propanoanthracen-12-one* (**7**). To a solution of SmI_2_ (2.1 g, 5.2 mmol) in THF (2 mL) was added solution of compound **6 **(0.75 g, 2.6 mmol) in THF (6 mL). The mixture was stirred at room temperature for 4 h, hexane was added , the mixture was filtered concentrated under vacuum. The crude product was purified by flash column chromatography on silica gel (petroleum ether-ethyl acetate 5:1) to give 7 (0.4 g, 56%) as a white solid, m.p. 128 ºC. IR (KBr): ν = 3070, 2912, 1683, 1475, 1448, 717cm^-1^; ^1^H-NMR δ 2.61 (s; 2H, H-13), 2.75 (d; *J* = 3.68 Hz, 2H, H-11), 3. 25 (d; *J* = 5.84 Hz, 2H, H-1'), 4.27 (t; *J* = 3.68 Hz, 1H, H-10), 5.15 (dd; *J* = 10.28, 1.84 Hz, 1H, H-3'), 5.30 (dd; *J* = 17.6, 1.84 Hz, 1H, H-3'), 5.69 (m; 1H, H-2'), 7.20–7.23 (m; 8H, aromatic-H); ^13^C-NMR δ 37.62, 43.56, 43.62, 50.26, 59.42, 118.25 134.60, 124.98, 126.31, 126.91, 127.11, 134.60, 140.11, 142.01, 208.88; MS (EI) *m/z* (%) = 274 (100) [M^+^], 275 (23), 231 (28), 217 (41), 216 (19), 215 (38), 203 (20), 202 (27), 191 (43), 189 (24); HRMS (EI) Calcd. for C_20_H_18_O [M^+^] 274.1359, Found 274.1358.

*9-(Prop-2-en-1-yl)-9,10-dihydro-9,10-propanoanthracene* (**8**). A mixture of ketone **7 **(0.88 g, 3.21 mmol), KOH (0.72 g, 12.83 mmol), hydrazine hydrate (2.285 g, 45.7 mmol) and triethyleneglycol (4 mL) was stirred at 150 ºC for 5 h. Then the water was removed by a Dean-Stark separator, and the reaction mixture was heated for a further 5 h to 200–210 ºC. After cooling to room temperature, the reaction mixture was treated with dil. HCl (until pH = 2 was reached). The aqueous layer was extracted with toluene, and the combined organic phases were washed with brine, dried with MgSO_4_ and concentrated under vacuum. The crude product was purified by flash column chromatography on silica gel (petroleum ether-ethyl acetate 5:1) to give **8** (0.63 g, 57%) as a yellow oil. IR (CDCl_3_): ν = 3068, 3016, 2958, 2926, 1473, 1452, 752 cm^-1^; ^1^H-NMR δ 1.25–1.29 (m; 2H, H-12), 1.31 (t; *J* = 6.6 Hz, 2H, H-13), 1.63 (t; *J* = 5.88, 2H, H-11), 3.19 (t; *J* = 2.58 Hz, 2H, H-1'), 3.99 (t; *J* = 3.68 Hz, 1H, H-10), 5.17 (m; 1H, H-3'), 5.27 (m; Hz, 1H, H-3'), 5.79 (m; 1H, H-2'), 7.22–7.29 (m; 8H, aromatic-H); ^13^C-NMR δ 23.53, 29.86, 39.22, 39.39, 45.71, 46.70, 117.42, 124.45, 126.08, 126.32, 126.37, 136.70, 143.25, 143.94; MS (EI) *m/z* (%) = 260 (61) [M^+^], 232 (19), 231 (42), 220 (27), 219 (85), 218 (55), 217 (53), 204 (18), 203 (53), 202 (60), 192 (29), 191 (100), 189 (61), 178 (44), 176 (15),165 (36), 152 (16); HRMS (EI) Calcd. for C_20_H_20_ [M^+^] 260.1563, Found 260.1565.

*2-(9,10-Dihydro)-9,10-propanoanthracen-9-yl)ethanal* (**9**). The tetracyclic alkene **8** (0.3 g, 1.15 mmol) was dissolved in CH_2_Cl_2_ (*ca.* 9 mL) and ozonolyzed at -78 ºC. After the reaction was complete (blue color), Me_2_S (6 equiv.) was added, and the reaction mixture was stirred for a further 4 h while it warmed to room temperature, and the volatile components were removed under vacuum. The crude product was purified by flash column chromatography on silica gel (petroleum ether-ethyl acetate 15:1) to give **9** (0.18 g, 60%) as a white solid, m.p. 94 ºC; IR (CDCl_3_): ν = 3064, 3018, 2931, 2856, 1728, 1477, 1452, 754 cm^-1^; ^1^H-NMR δ 1.22–1.26 (m; 2H, H-12), 1.67 (t; *J* = 4.4 Hz, 2H, H-13), 1.72–1.72 (m; 2H, H-11), 2.81 (dd; *J* = 16.88, 3.68 Hz, 1H, H-1'), 2.94 (dd; *J* = 16.88, 2.96 Hz, 1H, H-1'), 3.99 (t; *J* = 3.68 Hz, 1H, H-10), 6.96–7.23 (m; 8H, aromatic-H), 10.14 (s; H-C=O); ^13^C-NMR δ 22.10, 29.15, 37.84, 45.94, 46.53, 57.94, 126.08, 126.22, 126.37, 126.51, 142.59, 143.35, 202.48; MS (EI) *m/z* (%) = 262 (38) [M^+^], 234(37), 233(56), 220(44), 219(80), 218(67), 205(42), 204(26), 203(31), 202(32), 192(55), 191(100), 189(53), 178(44.85), 176(17), 165(33), 152(25); HRMS (EI) Calcd. for C_19_H_18_O [M^+^] 262.1359, Found 262.1358.

*2-(9,10-dihydro-9,10-propanoanthracen-9-yl)-N-methylethanamine* (**2**). Titanium(IV) isopropoxide (0.1 mL, 0,25 mmol) was added to a commercially available solution of methylamine in methanol (2M, 7.5 mL) followed by the addition of the aldehyde **9** (0.22 mL, 0.22 mmol). The reaction mixture was stirred at ambient temperature for 4 h, after which sodium borohydride (7.7 mg, 0.19 mmol) was added and the resulting mixture was further stirred for another period of 4 h. The reaction was then quenched by the addition of water (0.1 mL), the resulting inorganic precipitate was filtered and washed with diethyl ether (2 mL). The organic layer was separated and the aqueous part was further extracted with diethyl ether (2 × 4 mL). The combined ether extracts were dried over K_2_CO_3_. Removal of the solvent under vacuum gave bishomobenzoctamine **2** in high purity (0.04 g, 57%) as a white viscous liquid. IR (CDCl_3_): ν = 3448, 3338, 2962, 2926, 2852, 1598, 1475, 1450, 1261, 1093, 1020, 800 cm^-1^; ^1^H-NMR δ 1.09 (t; *J* = 6.6 Hz, 2H, H-13), 1.19–1.62 (m, 6H, H1', H11, H-12), 2.24 (s; 3H, CH_3_), 2.51–2.28 (m; 2H, H-2'), 2.32 (s; 1H, NH), 3.97 (t; *J* = 4.4 Hz, 1H, H-10), 7.19–7.40 (m; 8H, aromatic-H); MS (EI) *m/z* (%) = 277 (33) [M^+^], 262 (33), 234 (16), 233 (26), 220(22), 219 (84), 218 (23), 205 (16), 203 (19), 202 (20), 192 (25), 191 (100), 189 (29), 178 (19); HRMS (EI) Calcd. for C_20_H_23_N [M^+^] 277.1829, Found 277.1830.

## 4. Conclusions

The above described sequence represents a successful seven-step synthesis of the bishomobenzoctamine **2**. The key cyclization step was accomplished through cycloaddition of α-bromoacrolein on 9-allylanthracene.
